# Book Review

**Published:** 1927

**Authors:** 


### Reviews of Books.

---

**Infections of the Hand.** By L. R. Fifield, F.R.C.S. Pp. 192. Illustrated. London: H. K. Lewis & Co., Ltd. 1926. Price 9s. 6d.—Kanavel's work on infections of the hand has brought to light many important facts relating to this most common, and too often inefficiently treated condition. The author of this book has condensed the work of Kanavel and others, and brought the whole to within reasonable limits; to be exact, 186 pages. The subject is presented in an orderly, co-ordinated, and readable fashion; no important fact is omitted, no superfluous matter included. The opening chapter deals with the anatomy of the fingers and hand, the important surgical features predominating. Chapter II. deals with whitlow followed by a few pages on the complications arising therefrom. Chapter IV. describes infections of the "Palm of the Hand," and the next their complications. Following these are descriptions of infections of the bones and joints, lymphatics, the dorsum of the hand, and, concluding, a chapter on prognosis and another on treatment. Perhaps the most prominent feature of the whole work is the excellence of the illustrations, some coloured plates, some photographs and many clear and excellent line drawings.

**Difficult Labour.** By Samuel J. Cameron, M.B., B.Ch. (Glas.), F.R.F.P.S.G. and John Hewitt, M.B. Pp. 305. 31 figures. London: Edward Arnold & Co. 1926. Price 10s. 6d. —This useful little manual deals with the management of the various obstetrical difficulties met with in practice, and embodies the methods of treatment carried out in the large maternity hospital to which both authors are attached. The subject matter is well arranged and lucidly expressed, while the teaching is sound, practical, and fully up-to-date. In the chapter dealing with the management of breech presentations hardly sufficient emphasis is laid on the need for slow delivery of the after-coming head to lessen the risk of foetal death from intracranial hæmorrhage caused by tears of the tentorium cerebelli. The authors strongly advocate the conservative treatment of concealed accidental antepartum hæmorrhage, which in all cases they treat with large doses of morphia followed by pituitrin, only resorting to cæsarean section if the patient shows no sign of improvement. The vaginal plug and abdominal binder they dismiss as useless in this condition. Only a few paragraphs are devoted to the treatment of labour complicated by cardiac disease, this important subject being dealt with very briefly, while operations for the enlargement of the pelvis are described at considerable length, a proceeding which hardly seems necessary in a book intended primarily for the general practitioner. The chapter dealing with rupture of the uterus and injuries to the birth canal is particularly good. This book should prove useful alike to practitioners and to students of medicine.

**Heliotherapy.** By A. ROLLIER, M.D. Translated by G. DE SWIETOCHOWSKI, M.D., M.R.C.S. Second edition. Illustrated. Pp. 318. Oxford Medical Publications. Price 20s.—The second edition of Dr. Rollier's book will be as much appreciated by the medical profession as was the first. It is a further record of his clinical experience, now extending over twenty years, and dealing with the therapeutic action of sunlight and Alpine air on disease, especially surgical tuberculosis. He claims that his method of treatment is capable of curing surgical tuberculosis in all its forms, and at all ages. Of special importance is the chapter dealing with dosage, technique, and clinical results. Dr. Rollier discusses very fully the question of dosage and lays particular emphasis on overdose, pointing out that it may lead to serious or fatal result. His account of splints and immobilising apparatus is easy to follow and contains many original ideas. The notes on cases, accompanied by many illustrations from photographs, afford convincing evidence of the great benefit to be derived from heliotherapy. The book is well produced and excellently illustrated.

**Wheeler's Handbook of Medicine.** By WILLIAM R. JACK, M.D. Eighth edition. Pp. 646. Edinburgh: E. and S. Livingstone. 1926. Price 12s. 6d.—The fact that this handbook has already run through eight editions and four reprints is sufficient evidence of the high esteem in which it is held. One is astonished at the amount of information contained within so small a compass, the arrangement of the subject matter is particularly good and well indexed. The book opens with two useful chapters upon general medical pathology. Modern clinical laboratory tests receive a full share of attention, this is particularly noticeable in the chapter on renal disease. There are a number of simple, useful and original diagrams in the chapter on diseases of the nervous system. Where so much is compressed into a small compass, it must necessarily follow that certain subjects receive somewhat inadequate treatment. This is perhaps most noticeable in the cases of functional dyspepsia and encephalitis lethargica. In the main, however, the book is excellent throughout.

**Hewat's Examination of the Urine.** Revised by G. L. MALCOLM SMITH, M.B., F.R.C.P. Ed. Seventh edition. Pp. viii. 228. Illustrated. Edinburgh: E. and S. Livingstone. 1926. Price 3s.—The object of this handbook is to provide a description of the clinical tests that can be carried out with the minimum amount of apparatus. It has been a favourite with generations of medical students and its reputation will be still further maintained by this new edition, revised and enlarged by Dr. Malcolm Smith. The chapters on urine, blood and fæces have been brought up-to-date and new sections have been added on Fractional Gastric Analysis, Renal Efficiency Tests, and Examination of the Cerebro-spinal Fluid.

**Surgical Applied Anatomy.** By Sir FREDERICK TREVES, Bart. Eighth edition. Revised by Professor C. C. CHOYCE, C.M.G., C.B.E., M.D., F.R.C.S. London: Cassell & Co., Ltd. Pp. 727. 162 illustrations. Price 14s. net.—One takes up the new edition of this well-known book with a feeling that the reviewing of it will be an easy and pleasant task: it was familiar before, and the revision has been carried out by a well known teacher of Surgery. While it is not a very common thing for a students' text-book to reach its eighth edition, it must be still less so for a book to approach the jubilee of its first appearance; it is well over forty years since the first edition saw the light. It may be said at once that the revision has been carried out, as one would expect, in a thorough and careful way, the result being that the book should prove as valuable as it has done in the past. The senior student, unfortunately, has a rooted objection to the idea of learning any more anatomy: he does not realise that he has here a book which will help him to learn and remember the essential facts of the subject in the easiest and pleasantest way. If only taken up at odd moments, and opened at random, it will well repay perusal even in this casual fashion. We can find only a misprint or two, and no important omission. Perhaps a reference might be inserted to the method of doing gastro-jejunostomy which is now in increasing favour, by bringing both the viscera out through an aperture made in the gastro-colic omentum, as well as the usual one through the transverse mesocolon. This excellent method is not too well known to students, and the study of the anatomy of the procedure affords a useful mental exercise. We heartily commend the present edition to students in the confident hope that it will serve them as faithfully as it has done their predecessors.

**A Shorter Surgery.** By R. J. McNeill Love, M.S., F.R.C.S. Pp. 298. 43 illustrations. London: H. K. Lewis & Co., Ltd. Price 12s. 6d.—Many are the works now available, written expressly with the purpose of extracting from the wide field of surgery just such essentials as will enable the student to surmount his qualifying examinations. This Short Surgery adds yet one more to the list. The volume contains nothing novel in its composition, but so far as the reader is content with the material it contains, it is good. Every chapter is concise, well arranged, and the illustrations are clear and instructive. The classifications, though somewhat lacking in the bare essentials of appended explanations, are simple and in agreement with modern teaching. Such omissions as the causal organisms in empyema and classification of cerebral and spinal tumours might be remedied with benefit. We consider that short notes on the treatment of such conditions as burns and carbuncles would be useful, and that both might be added to the index. Altogether the book is admirably suited to the purpose for which it is written.

**Materia Medica and Therapeutics.** By J. Mitchell Bruce, C.V.O., M.D., F.R.C.P. and Walter J. Dilling, M.B., Ch.B., Thirteenth edition. Pp. xv., 686. London: Cassell & Co., Ltd. 1926. Price 10s. 6d. net.—It is over forty years since the first edition of "Mitchell Bruce" was published, and during these years it has been a popular book on what is, unfortunately, with medical students an unpopular subject. The present edition is written in conjunction with Professor W. J. Dilling. It deals in a simple way with the principles of Materia Medica in the true sense of the phrase, and, therefore, includes an account of a vast number of drugs, and describes their preparation, action and clinical use. If any criticism of this standard work can be made it is that too much space is given to description of the appearance of crude drugs, which is hardly necessary for medical students. Again, constitutional formulæ are more easily remembered than molecular formulæ. Apart from such minor antiquities, the book is well up-to-date. There is a clear and reasonable account of insulin, and reference is made to sympathetic stimulants and depressants. Useful sections are those devoted to Electro-therapeutics, Massage, Exercises, and Invalid Diet. We heartily commend this classic work.

**Diseases of the Heart and Lungs, for Nurses.** By A. I. G. McLaughlin, M.B. Pp. 185. Illustrated. London: Faber and Gwyer, Ltd. 1926. Price 4s. 6d.—This is a small book which should prove extremely valuable, not only to nurses, for whom it is primarily written, but also to medical students needing a clear and concise introduction to diseases of these organs. The chapters on anatomy and physiology are well illustrated and all the main facts clearly and briefly given. The essentials of nursing treatment are admirably portrayed, whilst modern methods of medical and surgical treatment, such as Artificial Pneumothorax and the injection of Lipoidol are introduced. In the various diets given, there appears to be no mention of the value of fresh fruit and fruit juices. The book has a complete index, is handy and concise. The clear statement of the essentials of nursing treatment is most valuable.

**Types of Mind and Body.** By E. Miller, M.B., M.R.C.S. Pp. 132. London: Kegan, Paul, Trench, Trubner & Co., Ltd. 1926. Price 2s. 6d.—In this valuable addition to an admirable series of essays on medical subjects, Dr. Miller has succeeded in presenting clearly and succinctly the recent views on mental and bodily types. He starts with morphological types, the most important contribution to the study of which is Kretchmer's classification into pyknic and asthenic types. Physiological differences are discussed with a short survey of the modern knowledge of the function of the endocrine organs and of the sympathetic and parasympathetic nervous systems. A warning is given that enthusiasm for this approach to the study of personality types should be tempered with discretion. On the psychological side, it is justly pointed out that any recognised types can only represent tendencies and not states of the personality, and special attention is given to the cyclothyme and schizothyme personalities, and their correspondence to the pyknic and asthenic types of bodily development. Finally, it is pointed out how the cross-currents of individual experience and conflict may modify the expected behaviour of even definite examples of the above types.

**Light Treatment in Surgery.** By Dr. O. BERNHARD (St. Moritz). Translated by R. KING BROWN, M.D. Pp. xii., 317. Illustrated. London : E. Arnold & Co. 1926. Price 21st. net. —This book consists of two parts of very unequal merit. The first part, dealing with the history and general principles of light treatment, is full of interesting information. It is simply and clearly written and should be in the hands of anyone who wishes to go in for heliotherapy. The second part deals with special conditions, particularly surgical tuberculosis. There is too little detail to make it of much use to those who wish to undertake this form of treatment in Great Britain. In this country our sunlight is such an uncertain quantity that it must be supplemented by artificial means to a much greater extent than is necessary in the high Alps. Here, too, we rarely need to adopt the elaborate means for avoiding sunburn which are so essential in places where the atmosphere is less dense, and filters off fewer of the chemical rays. Our cures are slower, and splinting, extensions and apparatus are necessary adjuvants to treatment upon the use of which Dr. Bernhard scarcely touches. The book is well printed, and there are few typographical errors. Some of the photographs are poor and fail to show what they are intended to demonstrate. Dr. R. King Brown is to be congratulated upon his very excellent translation.

**Fundamentals of the Art of Surgery.** By JOHN H. WATSON, M.B., B.S., F.R.C.S. Pp. x., 349. Illustrated. London : Wm. Heinemann, Ltd. 1926. Price 17s. 6d. net.—The drama of the operating theatre is often enacted and its story is ably written in surgical literature so that no student need be ignorant of it. Instruction is, however, apt to be scamped in the simpler steps of preparation for the great event, and the labours of tending the wound and nursing the patient back to usefulness after a surgical operation. The student learns too little of the numerous painful pitfalls that await the surgeon when considering the risks an operation entails for his patient. Some of these may be merely irritating, but others are fraught with disaster. He is apt to gain a biased opinion as to the appropriateness of operative procedures in different patients, as in the practice of a good surgeon the unsuitable cases are sifted before they reach the operating table. *Fundamentals of the Art of Surgery* is a veritable insurance against misapplied surgical intervention. Likewise, for the anxious doctor during the stormy days that may face the patient after the operation, this book is a sure and ample guide. Clear and detailed description of the pre- and post-operative care of the patient is a sore need well met by this pioneer literary venture. The author has conferred a boon on the profession, and is to be heartily congratulated on combining with a scientific presentation of his subject good style and loftiness of aim.

**Clinical Examination of the Nervous System.** By G. H. MONRAD-KROHN, M.D., M.R.C.P. Third edition. Pp. xvi., 201. Illustrated. London: H. K. Lewis & Co., Ltd. 1926. Price 7s. 6d.—As a guide to the complete examination of the nervous system this little book is admirable. The author describes very clearly the different methods of examination of the various parts of the nervous system, including the examination of the autonomic system. An outline of the examination of the mental state is given, whilst a short description of the measurement of intelligence in children, cistern puncture, and other new clinical methods are also included. The book is copiously illustrated, and the illustrations are well chosen, some of them being reproductions from photographs, others diagrammatic, and all help to elucidate the text. Nineteen new illustrations have been added since the last edition. Some routine method is necessary in all neurological examinations, and this book forms a most useful explanatory guide.

**Medical Case Taking.** By A. M. KENNEDY, M.D. Pp. 148. London: Edward Arnold & Co. 1926. Price 5s.—This book is intended to help the student in becoming conversant with his work in the medical wards. Its appearance at the present time, when the student has so many duties to perform outside the wards, in completing his curriculum before qualification, would seem to be very opportune, and the author loses no time in pointing out the necessity of gaining first-hand experience and practice at the bedside. He points out the responsibility of the student in regard to the patient and the actual use of notes efficiently taken, with their object, both as regards future reference and in giving practice and confidence to the student which should stand him in good stead when he reaches the examination room. There is no doubt that the average student regards note taking as a bore of doubtful utility, and develops a sort of note-taking phobia, with the result that he becomes mildly neurasthenic on approaching his " long case " in his final, does badly in marshalling his facts on paper for the examiner, and loses many marks in consequence. The remarks in regard to family history, social and working surroundings of the patient might seem rather cumbrous and likely to discourage the student in this sphere of his investigations by conveying to him an impression of lack of definition and conciseness in this part of his task. The chapter on " The Present Condition " shows how valuable clues may be gained by a close superficial scrutiny, and the examination of a nerve case, the *bête noire* of many, is carefully described. This is a book which is not intended to supplant works on general medicine, but will be helpful to the student in his consultations with his patient, in his associations with his chief, and in his efforts with his examiner.

**Dental Prosthetics (Outlines of Dental Science IV.).** By J. D. LOGAN, L.D.S. Pp. 219. Illustrated. Edinburgh: E. and S. Livingstone. 1926. Price 7s. 6d.—The author is ambitious. In his preface he states that his object is to describe within the smallest space what he considers to be the principles of denture construction, and he hopes as a result to enable the student to become a skilful artificer. We are doubtful of the value of "outlines," though they may serve as an introduction to the subjects dealt with. In this way the present volume can be recommended to students beginning their course in Dental Prosthetics. It describes the main principles, but unfortunately several favourite examination tests, such as backing and soldering flat teeth, fitting of tube teeth, and the making of regulating appliances are not described.

**X-Ray Therapy.** By Dr. R. LENK (Vienna). Translated by T. I. CANDY, M.B. Pp. 119. Oxford University Press. 1926. Price 6s. 6d.—We have, in this hand-book, a concise summary of the indications and prognosis of X-ray therapy as practised and approved by the " moderate " Vienna school under Professor Holzknecht. Several things should strike the English reader. The number of disorders, quite apart from malignant disease, which react satisfactorily to suitable X-ray dosage; the abolition of any fear of X-ray "burns" under modern technique; and the extraordinary value of X-rays in treating disorders of menstruation and allied conditions. The book has already been translated into five languages. It is primarily written for the general practitioner, who can see at a glance from the Holzknecht "formula" introduced under each disease, the probable duration of treatment, number of "sittings," etc., information which his patient often wants to know before being sent to the practising radiologist. We congratulate Dr. Candy on his translation of a book that ought to be most valuable, both in private practice and also in the treatment department of a hospital.

**High Blood Pressure: Its Varieties and Control.** By J. F. HALLS DALLY, M.A., M.D., B.Ch., M.R.C.P. Second Edition. Pp. xvi., 196. London: Wm. Heinemann Ltd. 1926. Price 12s. 6d.—This hand-book, the first edition of which we reviewed about three years ago, has now been revised so as to include some notice of the more recent work on the subject. The new matter includes an account of the behaviour of blood pressure in pulmonary tuberculosis, and notes on the value of balneology and physiotherapy in the treatment of hyperpiesis.

**The Pathology and Treatment of Diabetes Mellitus.** By GEORGE GRAHAM, M.A., M.D., F.R.C.P. Second Edition. Pp. xv., 230. Oxford Medical Publications. 1926. Price 8s. 6d.—The second edition of Dr. Graham's book on Diabetes has undergone many alterations in order that it might embody the new light which the discovery of insulin has thrown on the subject. Dr. Graham's account of the pathology of Diabetes Mellitus and the various problems of carbohydrate metabolism is excellent. When, however, it comes to treatment, Dr. Graham still advocates a considerable degree of undernutrition. The "Ladder-diet" of St. Bartholomew's Hospital appears to be based on a misgiving whether adequate insulin with adequate carbohydrate affords the same degree of rest to the island-cells of Langerhans as does inadequate carbohydrate with stinted insulin. Increasing clinical experience will settle the question, in the meantime it suffices to point out that the divergence of opinion exists. Furthermore, it should be observed that the Toronto School is emphatic in its preference for adequate carbohydrate and ample insulin.

